# Modified xiaoyao san combined with chemotherapy for breast cancer: A systematic review and meta-analysis of randomized controlled trials

**DOI:** 10.3389/fonc.2023.1050337

**Published:** 2023-03-22

**Authors:** Junhua Pan, Shunlian Fu, Qian Zhou, Dajun Lin, Qiu Chen

**Affiliations:** Hospital of Chengdu University of Traditional Chinese Medicine, Chengdu, China

**Keywords:** Chinese herbal medicine, breast cancer, meta - analysis, xiaoyao san, chemotherapy

## Abstract

**Background:**

Breast cancer is a common cause of cancer-related death worldwide. Chemotherapy plays an indispensable role in the conventional treatment of breast cancer, bringing some physical burdens and discomfort on cancer patients. Consequently, more and more patients turn to seeking the help of Complementary and Alternative Medicine (CAM), mainly traditional Chinese medicine (TCM). Xiaoyao san (XYS), a classical formula, has been shown to improve symptoms of breast cancer. An increasing number of researches suggest that compared to chemotherapy alone, Chinese herbal medicine combined with chemotherapy could increase effectiveness and reduce toxicity caused by chemotherapy. Emerging experimental research continuously demonstrated some of the components in XYS could stop breast cancer tumor cells from growing. However, the efficacy and safety of modified XYS combined with chemotherapy remain to be determined. Therefore, it is essential to evaluate the comparative effectiveness and safety of modified XYS combined with chemotherapy in-depth, thus providing clinicians and policymakers with evidence-based guidance and new treatment options.

**Objective:**

To comprehensively evaluate the efficacy and safety of modified XYS in conjunction with chemotherapy in treating breast cancer by conducting a meta-analysis.

**Methods:**

8 databases were systemically searched until April 3, 2022, including Web of Science PubMed, EMBASE, Cochrane Library, China National Knowledge Infrastructure (CNKI), Wanfang, Chinese Scientific Journals Database (VIP), and Chinese Biological Medical Database (CBM). Relevant randomized controlled trials (RCTs) comparing modified XYS in combination with chemotherapy versus chemotherapy alone were included. For the evaluation of methodological quality, Cochrane Collaboration was considered. Software Review Manager (version 5.4) was used for data analysis. Software STATA (version 15.0) was employed for sensitivity analysis and publication bias.

**Results:**

Altogether, 17 RCTs involving 1207 patients were investigated in the current review. The findings revealed that modified XYS combined with chemotherapy could lead to beneficial improvements compared to chemotherapy alone. More specifically, the combined therapy could enhance the short-term efficacy in the treatment of solid tumors (OR: 1.74; 95% CI 1.27 to 2.39; P = 0.0006; I^2^ = 0%); improve QOL (quality of life) (OR: 3.75; 95% CI 2.58 to 5.44; P < 0.00001; I^2^ = 0%); reduce clinical symptoms (OR: 3.69; 95% CI 1.43 to 9.49; P = 0.007; I^2^ = 53%); ease depression (MD: -12.96; 95% CI -16.09 to -9.83; P < 0.00001; I^2^ = 0%); increase leukocytes (OR: 0.32; 95% CI 0.20 to 0.50; P < 0.00001; I^2^ = 0%) and platelets (OR: 0.37; 95% CI 0.20 to 0.67; P = 0.001; I^2^ = 0%); reduce nausea and vomiting (OR: 0.26; 95% CI 0.15 to 0.44; P < 0. 00001; I^2^ = 0%); mitigate cardiotoxicity (OR: 0.16; 95% CI 0.07 to 0.36; P<0.00001; I^2^ = 0%); prolong survival time (OR: 2.19; 95% CI 1.03 to 4.66; P = 0.04; I^2^ = 0%), compared to chemotherapy alone. Unfortunately, there was no statistically significant difference in damage to the liver and kidney (OR: 0.59; 95% CI 0.29 to 1.21; P = 0.15; I^2^ = 0%).

**Conclusion:**

The existing evidence suggests modified XYS combined with chemotherapy leads to beneficial improvements in the management of breast cancer, which may serve as a promising therapy for breast cancer in clinical practice. Given the limited number of high quality RCTs, more rigorous, scientific, double-blinded, large-scale, multi-center clinical trials are warranted further.

**Systematic review registration:**

https://www.crd.york.ac.uk/PROSPERO/, identifier CRD42022357860.

## Introduction

Breast cancer remains a major public-health problem now (1–3). Breast cancer, the most common female malignancy worldwide, accounts for 23% of all cancers and 14% of cancer-related deaths (4, 5). In China, more than 169,000 women suffer from breast cancer every year (6). In recent years, the incidence rate and mortality of female breast cancer in China are on the rise (7). Chemotherapy plays a crucial role in the management of breast cancer, having been extensively used in combination with surgery, radiation therapy, and other conventional treatments (8). However, chemotherapy also brings along with some discomforts and physical burdens (8, 9). Therefore, there is a compelling need to update the treatment, which could overcome these drawbacks.

Patients with breast cancer gradually turn to alternative therapy, notably complementary and alternative medicine (CAM), for symptom alleviation (10). With the increasing acceptability of CAM among cancer patients, traditional Chinese medicine (TCM) has become the main component of CAM modality (11). TCM, mainly Chinese Herbal Medicine (CHM), has received incredible popularity in China, some other Asian countries as well as western countries (12). CHM combined with chemotherapy has shown remarkable potency in treating various cancer, including lung cancer, breast cancer, prostate cancer, and Pancreatic cancer (13–16). XYS was first mentioned in the Song Dynasty’s Tai Ping Hui Min He Ji Ju Fang (Formulas of the Administration of People’s Welfare Pharmacy). This prescription contains the following 8 herbs: Chai Hu (*Radix Bupleuri*), Dang Gui (*Angelicae Sinensis Radix*), Shao Yao (*Paeoniae Radix Alba*), Fu Ling (*Poria*), Bai Zhu (*Macrocephalae Rhizoma*), Gan Cao (*Radix Rhizoma Glycyrrhizae*), Bo He (*Menthae Haplocalycis Herba*), Sheng Jiang (*Zingiberis Rhizoma*). The primary function is to soothe the liver and disperse depression, strengthen the spleen and tonify the blood Unfortunately, XYS in combination with chemotherapy lacks evidence-based support. As a matter of fact, there are three meta-analyses of CHM in combination with chemotherapy for breast cancer, but no specific formula is suggested (14, 17, 18). Nonetheless, to our knowledge, this review is the latest on this topic. A systematic review and meta-analysis to evaluate the efficacy of XYS combined with chemotherapy in breast cancer has not yet been conducted. Therefore, we assess the efficacy and safety of modified XYS combined with chemotherapy in the treatment of breast cancer to provide Clinicians and health policymakers with convincing evidence.

## Methods

The Preferred Reporting Items for Systematic Reviews and Meta-analysis Statement (PRISMA) was used for this meta-analysis (19). This review was registered in PROSPERO as CRD42022357860. This review on modified XYS in the treatment of breast cancer was conducted on the basis of published articles, and no privacy issues were involved. Therefore, there were no ethical approval and privacy issues required.

## Search strategy

Relevant studies were retrieved from 8 electronic databases from their inception until April 3, 2022, including Web of Science PubMed, EMBASE, Cochrane Library, China National Knowledge Infrastructure (CNKI), Wanfang, Chinese Scientific Journals Database (VIP), and Chinese Biological Medical Database (CBM). There were no limitations on publication language. In literature retrieval, the strategy of subject words combined with free words were employed. To avoid omissions, we would manually check the reference lists of included articles to search for additional studies. The following items, “breast cancer,” “xiaoyao san,” and “chemotherapy,” were retrieved in the databases. A more detailed search strategy could be obtained in the **Supplementary Materials**.

## Selection criteria

The initial browse of titles and abstracts was separately searched by two researchers. Based on the titles and abstracts, animal studies, case reports, reviews, experience introductions, or non-randomized controlled trials were ignored. Interventions that were obviously inconsistent with our topic were not considered as well. A second browse was carried out as a full-text screening by two researchers independently. Based on the full-text, the chosen studies were rigorously screened under the inclusion criteria. When encountering a dispute, a third party would step in the argument. Any disagreements would be resolved by consultation. In accordance with the PICO criteria, the inclusion criteria of this analysis were formulated from four aspects: participant, intervention, control, and outcome.

### Type of studies

The relevant RCTs were included in this review. Non-randomized controlled trials were excluded.

### Type of participants

All eligible Patients must be definitely diagnosed with breast cancer either through pathology or cytology, regardless of the stage of cancer, gender, age, race, and severity.

### Interventions of experiment group

Various forms (i.e, decoction, powder, tablets) of XYS or modified XYS combined with chemotherapy.

### Interventions of the control group

Chemotherapy alone. The specific drugs and dosage of chemotherapy were unrestricted.

### Outcome measures

Primary outcome measures: the short-term efficacy in the treatment of solid tumors, and QOL. Secondary outcomes: clinical symptoms, depression, leukocyte, platelets, nausea and vomiting, damage to the liver and kidney, cardiotoxicity, survival rate, and at least one measure.

The exclusion criteria were as follows:

1. Repetitive articles.
2. Missing outcome measures.
3. Non-clinical RCTs.
4. Not XYS-categorized formulas.
5. The patients included in the review had serious organic diseases such as liver and kidney.
6. Non-human or animal studies.

### Data abstraction

According to the inclusion and exclusion criteria outlined above, the data were screened and extracted independently by two researchers. Afterward, the following information from each included RCT was extracted for inclusion: The first author, publication time, sample size, treatment method, type of breast cancer, estrogen receptor, tumor spread, tumor stage, age, average age, intervention, duration, and outcome measures were all extracted. When two researchers disagreed, a third author intervened to evaluate. Any dispute would be settled through negotiating or discussing within a group. The corresponding author reviewed the final data.

### Quality assessment

The quality of the study was rated independently by two reviewers according to the Cochrane collaboration tool (20). The assessment tools consist of the following 6 domains: random sequence generation (selection bias), allocation concealment (selection bias), blinding of participants and personnel (performance bias), blinding of outcome assessment (detection bias), incomplete outcome data (attrition bias), selective reporting (reporting bias), and other bias. According to how well the evaluation items above performed, the judgment of low risk, unclear risk, and high risk were given in turn. When two researchers disagreed, a third researcher intervened to evaluate.

### Statistical analysis

After data extraction, meta-analysis was performed by the statistical software Review Manager (version 5.4.1). The short-term efficacy in the treatment of solid tumors, QOL, clinical symptoms, leukocytes, platelets, nausea and vomiting, the damage to the liver and kidney, cardiotoxicity, and survival rate were regarded as dichotomous data, which were expressed as Odds Ratio (OR) for there variables; only depression was regarded as continuous data, which calculated mean differences (MD) as effect size, both with 95% confidence intervals (CI). The χ2 test and inconsistency index statistic (I^2^) were used to determine the magnitude of heterogeneity, thus determining the corresponding effects model. I^2^ < 25%, indicated low heterogeneity; I^2^ < 50%, indicates moderate heterogeneity; I^2^ > 50%, indicates high heterogeneity. If there was significant heterogeneity (I^2^ > 50% or P < 0.5), a random effect model was adopted; on the contrary, a fixed effect model was employed. For p < 0.05, T Pooled results were deemed statistically significant. If the included study had significant heterogeneity, sensitivity analysis or subgroup analysis was then performed to search for any potential sources of heterogeneity. When more than 10 studies are included, the funnel plot is drawn to identify the publication bias. If the funnel chart is symmetrical, there is no evidence of publication bias. To avoid subjectivity, Egger’s test is carried out if necessary.

## Results

### Study selection

**Figure 1:**
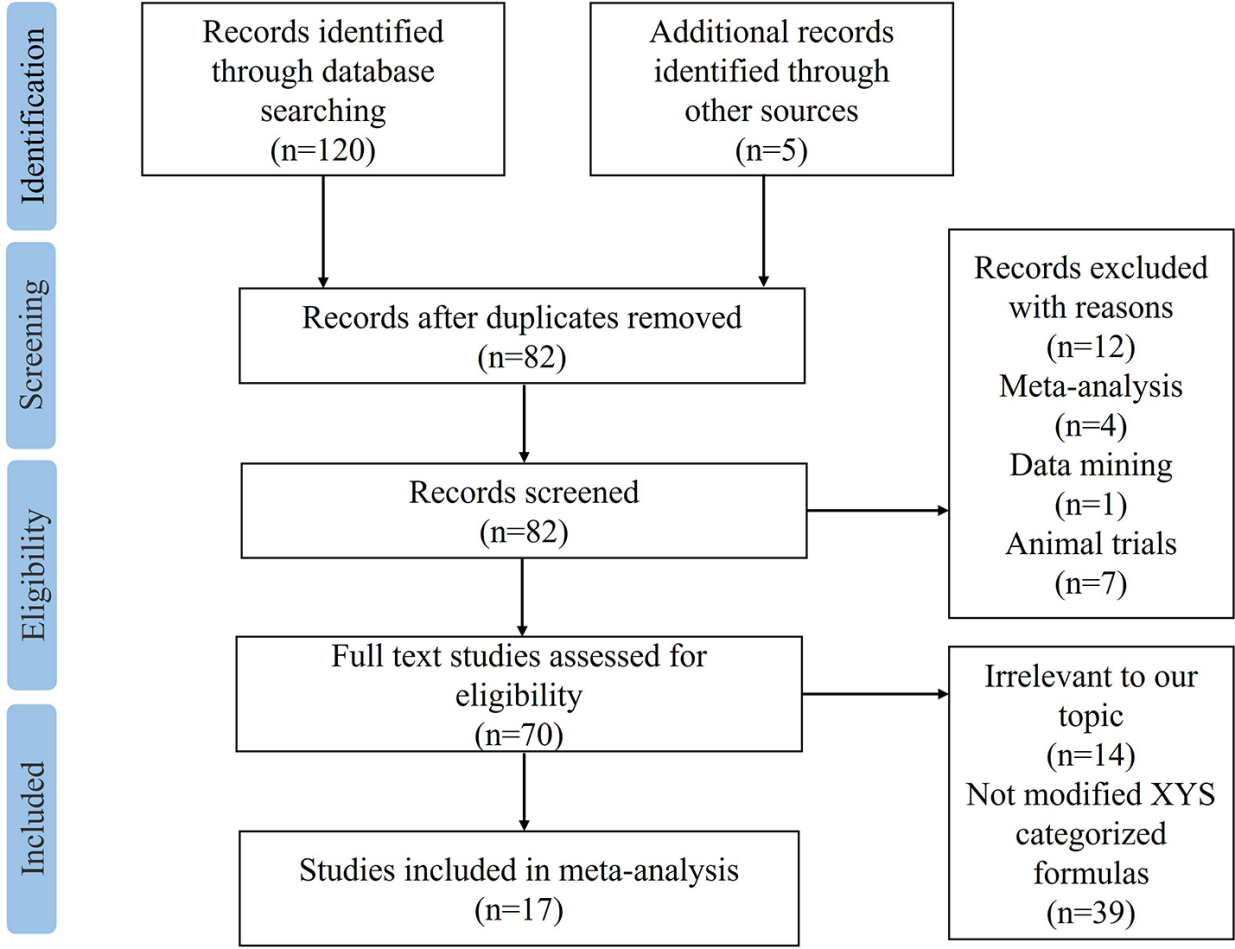
PRISMA flow chart.

A total of 120 studies were identified by searching the 8 electronic databases, among which 43 duplicated records were excluded. 5 articles were manually retrieved from other sources. After screening titles and abstracts, 12 studies were removed for not meeting the predefined criteria: 4 were meta-analyses, 7 were animal trials, and 1 was based on data mining. There were 70 studies left for further full-texts reading. Among the 53 excluded records, 14 were irrelevant to our topic, and 39 were not modified XYS-categorized formulas. Eventually, 17 eligible RCTs (21–37) were included in the final meta-analysis, with the detailed flow chart of study selection shown in **Figure 1**.

### Study characteristics

From 2002 to 2020, the baseline Characteristics of 17 RCTs enrolled in this study are summarized in **Table 1**. All studies were performed in mainland China and published in Chinese. 13 out of the enrolled studies received modified XYS combined with chemotherapy, while the remaining 4 studies used XYS combined with chemotherapy. The duration of treatment lasted from 6 to 36 weeks, with a single sample size of 40 to 120. The stage of breast cancer ranged from I to IV. Only 9 studies mentioned the specific type of breast cancer. 12 studies mentioned tumor spread. 3 studies claimed the estrogen receptor status were negative, the others remaining unclear. Patients across the studies did not share the same chemotherapy drugs. The most common collocation was FAC or capecitabine plus modified XYS, among which both made three times appearances. More details could be obtained in **Table 1**.

**Table 1:** Baseline Characteristics of 17 RCTs included in this meta-analysis.

Studies	Sample size(T/C)	Type of BC (number of cases)	Tumor spread	ERS	Diseases stage	Age/Average age (years)	Intervention measures	Duration (week)	Outcome measures
Jin et al. 2020 (17)	100(50/50)	–	–	–	–	T 25~75/50.56±2.49 C 23~74/51.53±8.64	Conventional chemotherapy+modified XYS/Conventional chemotherapy	6	④⑤⑦⑨
Lu 2019 (18)	80(40/40)	–	–	–	–	T 32~67/54.6±4.50 C 33~69/54.2±4.60	FAC+modified XYS/FAC	16	②
Li 2017 (19)	120(60/60)	Adenocarcinoma (34); Squamous cell carcinoma (29); Intraductal carcinoma (20); Papillary carcinoma (23); Simple carcinoma (14)	Yes	–	II: 23 III: 52 IV: 45	T -/50.14±6.21 C -/49.65±5.28	FAC+modified XYS/FAC	16	①②⑨
Xu et al. 2016 (20)	73(37/36)	–	Yes	–	IV: 73	T -/56.30±2.20 C -/55.40±1.90	GC+modified XYS/GC	6	①②③
Sun et al. 2016 (21)	64(32/32)	Invasive breast cancer (64)	Yes	–	I~III: 58 IV: 6	T 45.90±11.70 C 46.4±12.30	TE+modified XYS/TE	6	②④⑥⑦⑧⑨
Yang 2016 (22)	79(40/39)	Triple negative breast cancer (79)	No	(-)	–	T -\63.50±6.40 C -\62.20±6.20	Capecitabine+XYS/Capecitabine	12	①⑥⑦⑧
Du et al. 2015 (23)	60(30/30)	Adenocarcinoma (15); Squamous cell carcinoma (11); Simple carcinoma (11); Intraductal carcinoma (13); Medullary carcinoma (5); Papillary carcinoma (5)	–	–	IIA: 22 IIB: 20 IIIA: 18	T 30~69/50.3 C 29~70/50.5	FAC+modified XYS/FAC	16	②③⑩
Wang 2015 (24)	80(40/40)	Triple negative breast cancer (80)	Yes	(-)	III: 51 IV: 29	T 56~74/66.30±4.60 C 57~75/66.70±4.90	Capecitabine+XYS/Capecitabine	8	①⑩
Yang et al. 2015 (25)	42(21/21)	–	Yes	–	–	T 35~69/46.70±5.60 C 36~68/47.10±4.30	Teggio+XYS/Teggio	6	①
Zhu 2014 (26)	92(46/46)	Triple negative breast cancer (92)	Yes	(-)	–	T 55~75/62 C 55~75/65	Capecitabine+modified XYS/Capecitabine	12	①⑩
Sun et al. 2014 (27)	42(21/21)	–	–	–	I~IV:42	–	TE+modified XYS/TE	6	②⑤⑦⑧⑨
Sun et al. 2014 (28)	60(30/30)	–	Yes	–	IIIA: 14 IIIB: 10 IV: 6	T 32~65/46.50±6.60 C 35~67/47.30±5.70	CEF+Hei XYS/CEF	9	②⑤⑥⑦
Wang 2012 (29)	40(20/20)	Invasive ductal carcinoma (33); Invasive lobular carcinoma (4); Others (3)	Yes	–	IV: 40	T -/50.40±8.56 C-/52.60±9.00	GP+modified XYS/GP	6	①②③⑤⑥⑧
Huang et al. 2009 (30)	86(44/42)	Invasive ductal carcinoma (45); Simple carcinoma (16); Carcinoma (14); Highly differentiated mucous adenocarcinoma (8); Medullary carcinoma (3)	Yes	–	I~II: 86	25~82/53.5	CMF+ XYS/CMF	24-36	①②⑩
Wen et al. 2006 (31)	65(33/32)	Invasive ductal carcinoma (44); Simple carcinoma (11); Invasive lobular carcinoma (7); Intraductal carcinoma (3)	Yes	–	I: 3 IIA: 15 IIB: 34 IIIA: 9 IIIB: 4 IV: 6	T 26-75/48.61 C 27-73/48.75	CMF/CAF/NA+modified XYS /CMF/CAF/NA	18-24	①②⑤⑥⑦
Huang et al. 2003 (32)	62(32/30)	–	–	–	IIa: 14 IIa: 20 IIIa: 28	T 21~65/38.6 C 20~62/36.7	CMF+modified XYS/CMF	6	②③⑤⑦
Zhang 2002 (33)	62(32/30)	–	Yes	–	III: 45 IV: 17	T 30~62/48 C 31~64/47.5	CAF+modified XYS/CAF	6-8	①②⑤⑥⑨

T=experiment group, C=control group, ER=Estrogen Receptor Status,BC=Breast Cancer. FAC=Fluorouracil+Doxorubicin+Cyclophosphamide; TE=Taxotere+Epirubicin; GP=Gemcitabine+Cisplatin; CMF=cyclophosphamide+methotrexate+fluorouracil; CAF=Cyclophosphamide + Doxorubicin + 5-Fluorouracil; CEF=cyclophosphamide + epirubicin + 5-fluorouracil; NA=Norvinblastine+Epirubicin; GC=Gemcitabine+Cisplatin.

①The short-term Efficacy of solid tumors; ②Quality of Life (QOL); ③clinical symptoms; ④Depression; ⑤Leukocyte; ⑥platelets; ⑦nausea and vomiting; ⑧The damage to liver and kidney; ⑨Cardiotoxicity; ⑩survival rate.

### Assessment of methodological quality

**Figure 2:**
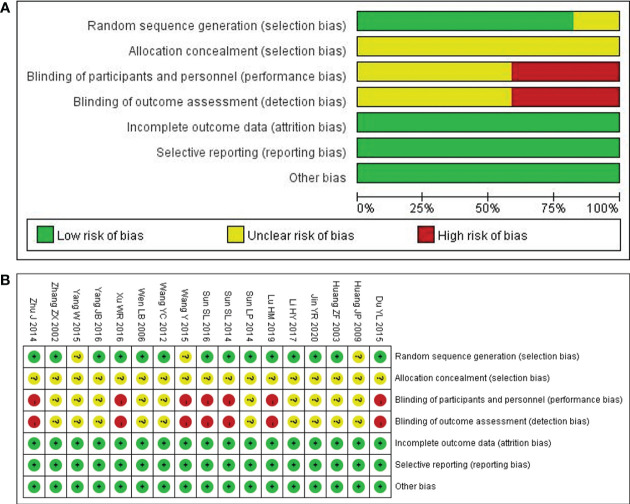
Quality assessment of the included comparative studies. **(A)** Risk of bias graph. **(B)** Risk of bias summary.

14 out of the included studies had mentioned that patients were randomly assigned to groups (20–33), while the remaining 3 did not mention that (28, 29, 34). Although 14 studies certainly claimed a random grouping, only 8 articles referred to the random number table (23–25, 31, 32, 35–37) with regard to allocation concealment, all the studies were judged as unclear for the lack of detailed information. Given blinding, 10 studies were assessed as unclear for insufficient information; 7 studies were judged as a high risk due to the non-blind method (22, 24, 25, 27, 28, 30, 31). None of the studies lost outcome data and reported follow-ups, resulting in a low risk of incomplete outcome data, selective reports, and other biases. In summary, the methodological quality was generally poor, as shown in **Figures 2A, B**. For a more comprehensive evaluation, GRADE (Grading of Recommendations, Assessment, Development, and Evaluations) was used to rate the evidence of quality as well. More detailed items are listed in **Table 2**.

**Table 2:** GRADE evidence profile.

Outcomes	RCTs	Quality assessment					No. of patients		Effect size	Quality
		Risk of bias	Inconsistency	Indirectness	Imprecision	Reporting bias	T	C		
The short-term efficacy of solid tumors	10	serious^a^	No	No	No	No	159/372	112/364	OR=1.74 (1.27 to 2.39)	Low
QOL	11	No	No	No	No	No	178/374	94/367	OR=3.75(2.58 to 5.44)	Moderate
Clinical Symptoms	4	No	serious^c^	No	serious^b^	No	96/119	66/116	MD=3.69 (1.43 to 9.49)	very low
Depression	3	No	No	No	serious^b^	No	103	103	OR=-12.96 (-16.09 to -9.83)	Low
Leukocyte	8	No	No	No	No	No	106/250	148/245	OR=0.32 (0.20 to 0.50)	Moderate
Platelets	4	No	No	No	serious^b^	No	63/115	84/112	OR=0.37 (0.20 to 0.67)	Low
Nausea and Vomiting	8	No	No	No	No	No	93/258	132/254	OR=0.26 (0.15 to 0.44)	Moderate
The Damage to Liver and Kidney	4	No	No	No	serious^b^	No	20/112	28/112	OR=0.59 (0.29 to 1.21)	Low
Cardiotoxicity	5	No	No	No	No	No	7/195	34/193	OR=0.16 (0.07 to 0.36)	Moderate
Survival Rate	3	No	No	No	No	No	116/128	101/124	OR=2.19 (1.03 to 4.66)	Moderate

a Most trials had unclear risk, and with high risk, but the result had good robustness. The evidence was rated down by only one level.

b The sample size was small and the confidence interval was wide. Therefore, the evidence was rated down by one level.

c Heterogeneity presented in them.Therefore, the evidence was rated down by one level.

### The short-term efficacy in the treatment of solid tumors

**Figure 3:**
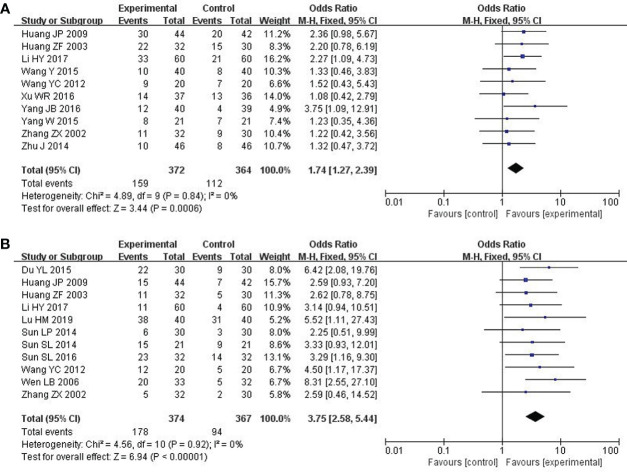
Forest plot of improvement in short-term efficacy of solid tumors and QOL. **(A)** The short-term efficacy in the treatment of solid tumors. **(B)** Quality of Life (QOL).

The RESIST (Response Evaluation Criteria in Solid Tumors) criteria, which were published in the European Journal of Cancer, were applied to evaluate the short-term efficacy in the treatment of solid tumors. The total effective rate is CR (complete response) plus PR (partial response). A total of 10 studies with 736 patients (23, 24, 26, 28–30, 33, 34, 36, 37) reported the short-term efficacy of modified XYS combined with chemotherapy in treating breast cancer. After the heterogeneity test (P = 0.84, I^2^ = 0%), so the fixed-effect model was considered. The improvements of modified XYS in short-term efficacy in treating solid tumors were more significant than that of chemotherapy alone (OR: 1.74; 95% CI 1.27 to 2.39; P = 0.0006; **Figure 3A**).

### QOL

QOL was assessed by Karnofsky score. 11 studies with 741 participants reported the QOL (22, 23, 25, 27, 31–37). After the heterogeneity test (P = 0.92, I^2^ = 0%), so the fixed effect model was adopted. The better improvements in QOL could be obtained in the combined group rather than chemotherapy alone (OR: 3.75; 95% CI 2.58 to 5.44; P < 0.00001; **Figure 3B**).

### Clinical symptoms

**Figure 4:**
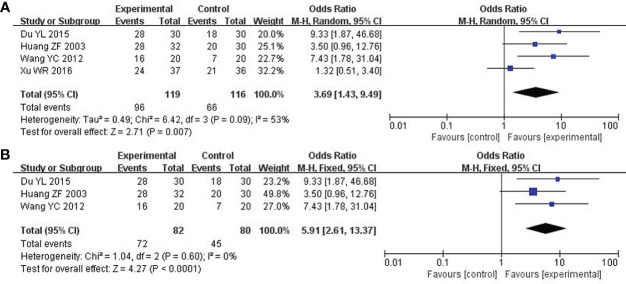
Forest plot of improvement in clinical symptoms. **(A)** Clinical symptoms. **(B)** Clinical symptoms (After removing the low quality study).

**Figure 5:**
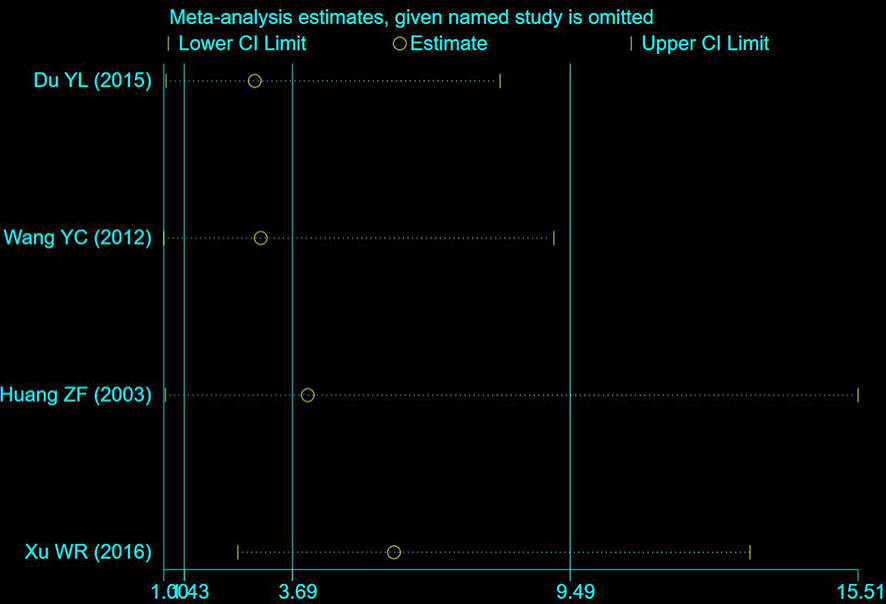
Sensitivity analysis on clinical symptoms.

A total of 4 studies including 235 participants had mentioned the clinical symptoms (24, 27, 33, 36). The clinical symptoms referred specifically to the improvement of clinical syndromes of TCM. However, moderate heterogeneity was measured (P = 0.09, I^2 =^ 53%), so the random effect model was employed. Compared to chemotherapy, the combined group was more efficient in alleviating the clinical symptoms (OR: 3.69; 95% CI 1.43 to 9.49; P = 0.007; **Figure 4A**). To explore potential sources of heterogeneity, sensitivity analysis was performed herein (**Figure 5**). By removing the study with low quality (20), there was no significant heterogeneity (P = 0.60, I^2^ = 0%). After removal, the pooled results were steady and unaltered, implying that the heterogeneity did not influence the results (OR: 5.91; 95% CI 2.61 to 13.37; P < 0.0001; **Figure 4B**). That was, partly, owing to the small sample size and low quality.

### Depression

**Figure 6:**
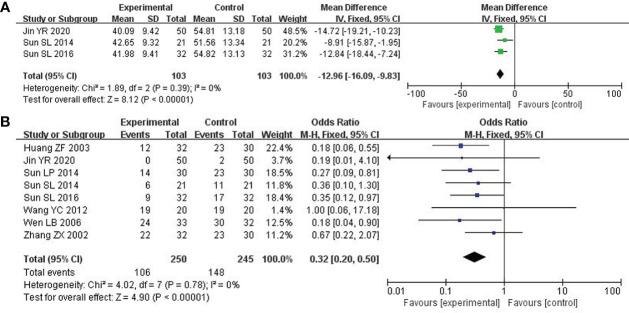
Forest plot of improvement in depression and Leukocyte. **(A)** Depression. **(B)** Leukocyte.

Depression was assessed by the Self-rating Anxiety Scale (SAS) score. 3 studies with 206 patients were investigated (21, 25, 31). After the heterogeneity test (P = 0.39, I^2^ = 0%), so the fixed-effect model was considered. The combined therapy could remarkably enhance the clinical efficacy in improving depression (MD: -12.96; 95% CI -16.09 to -9.83; P < 0.00001; **Figure 6A**).

### Leukocytes

8 studies including 495 participants reported the reduction of leukocytes (22, 25, 31–33, 35–37). After the heterogeneity test (P = 0.78, I^2^ = 0%), a fixed effect model was applied. Compared to the control group, the combined group was considerably effective in reducing leukocytes (OR: 0.32; 95% CI 0.20 to 0.50; P < 0.00001; **Figure 6B**).

### Platelets

**Figure 7:**
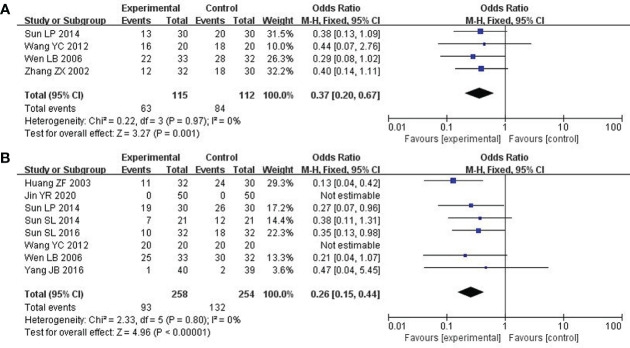
Forest plot of improvement in platelets and nausea and vomiting. **(A)** Platelets. **(B)** Nausea and vomiting.

4 out of included studies with 227 patients reported the platelets (32, 33, 35, 37). After the heterogeneity test (P = 0.97, I^2^ = 0%), a fixed effect model was adopted. In the improvement of platelets, the combined group surpassed chemotherapy alone (OR: 0.37; 95% CI 0.20 to 0.67; P = 0.001; **Figure 7A**).

### Nausea and vomiting

8 studies including 512 participants analyzed nausea and vomiting (21, 25, 26, 31–33, 35, 36). After the heterogeneity test (P = 0.80, I^2^ = 0%), a fixed effect model was used. Modified XYS combined with chemotherapy had few reports of nausea and vomiting over chemotherapy alone (OR: 0.26; 95% CI 0.15 to 0.44; P < 0. 00001; **Figure 7B**).

### The damage to liver and kidney

**Figure 8:**
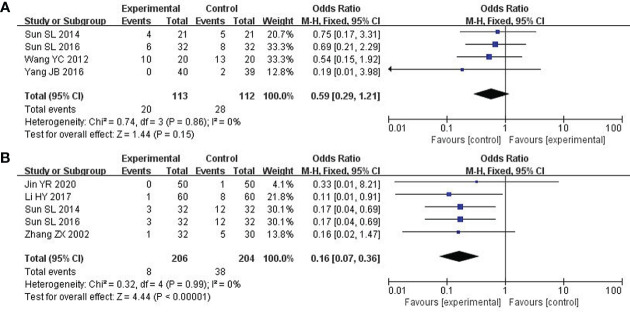
Forest plot of improvement in the damage to liver and kidney and Cardiotoxicity. **(A)** The damage to liver and kidney. **(B)** Cardiotoxicity.

4 studies about 225 patients reported damage to the liver and kidneys (25, 26, 31, 33). After the heterogeneity test (P = 0.86, I^2^ = 0%), so the fixed effect model was adopted. Unfortunately, there was no significant difference across these studies. Therefore, no definite conclusion could be drawn on the efficacy of modified XYS in reducing damage to the liver and kidney (OR: 0.59; 95% CI 0.29 to 1.21; P = 0.15; **Figure 8A**).

### Cardiotoxicity

5 studies including 410 participants reported cardiotoxicity (21, 23, 25, 31, 37). After the heterogeneity test (P = 0.99, I^2^ = 0%), so the fixed effect model was used. The findings displayed combined group was dramatically better than chemotherapy in reducing cardiotoxicity (OR: 0.16; 95% CI 0.07 to 0.36; P<0. 00001; **Figure 8B**).

### Survival rate

**Figure 9:**
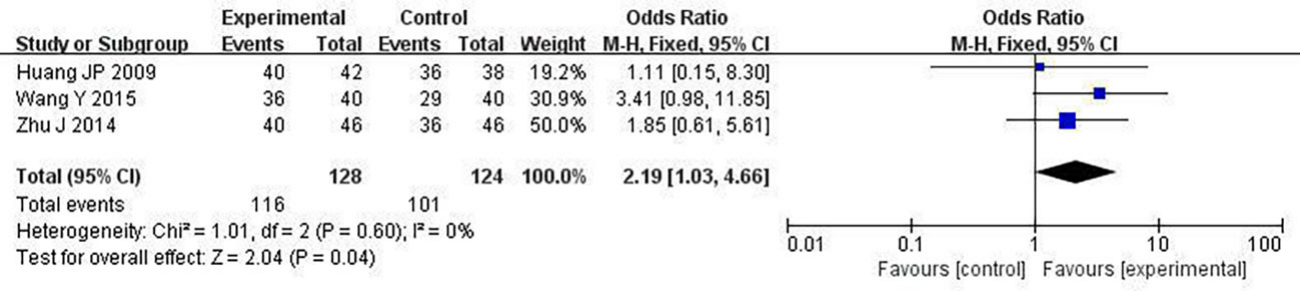
Forest plot of improvement in survival rate.

We chose one-year survival as a measure to report the survival rate. 3 studies including 252 patients analyzed one-year survival time (28, 30, 34). After the heterogeneity test (P = 0.60, I^2^ = 0%), so the fixed effect model was used. A better survival rate could be observed in the combined group (OR: 2.19; 95% CI 1.03 to 4.66; P = 0.04; **Figure 9**).

### Publication bias

**Figure 10:**
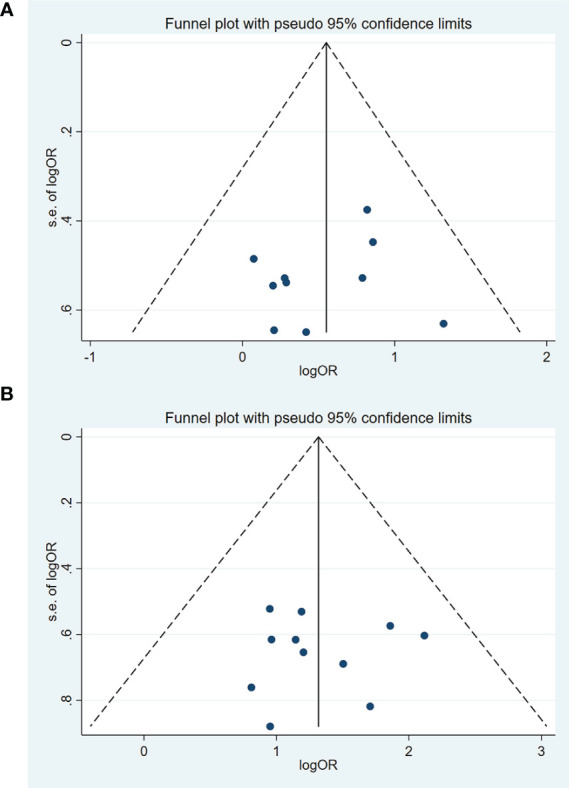
Funnel chart of publication bias in the short-term efficacy in the treatment of solid tumors and QOL. **(A)** The short-term efficacy in the treatment of solid tumors. **(B)** Quality of Life (QOL).

The funnel plots based on short-term efficacy in treating solid tumors and QOL were shown in **Figures 10A, B**. Judging from the figures, evidence of funnel plot symmetrically was observed, indicating no potential publication bias. To avoid subjectivity, P value of Egger’s test further demonstrated there was no publication bias about the short-term efficacy in treating solid tumors (P = 0.680) and QOL (P = 0.897).

## Discussion

Altogether, 17 RCTs involving 1207 patients were investigated in this review. The findings revealed that modified XYS combined with chemotherapy could lead to considerably beneficial improvements for breast cancer patients. Specifically, short-term efficacy in the treatment of solid tumors is better in modified XYS combined with chemotherapy. with regard to QOL, the combined therapy is superior to that of the chemotherapy. Additionally, the combined group is better than chemotherapy in eliminating the patients’ clinical symptoms. Nonetheless, high heterogeneity is identified. After removing the study with high heterogeneity, the results remain unaltered and robust with no heterogeneity. Meanwhile, depression in the combined group is discovered to be much lower than chemotherapy alone. The combined group results in a more favorable effect in leukocytes and platelets. Concerning nausea and vomiting, the combined therapy has few relevant reports. Compared to chemotherapy alone, XYS combined with chemotherapy is more effective in reducing cardiotoxicity. Moreover, a longer one-year survival time is in the combined group. Unfortunately, there was no obviously statistical difference in damage to the liver and kidney.

According to the statistics, there were 2.26 million incident breast cancer cases in 2020 globally (38). The incidence of breast cancer is nearly reaching 96%, which is only marginally less than that of lung cancer, and has been one of the cancers with tremendous growth in China over the past 30 years (39). Therefore, an adjuvant therapy or substitute to update the conventional treatment is urgently expected, meeting the unmet demand of cancer patients. Nowadays, the treatment of breast cancer includes surgery, chemotherapy, radiation therapy, hormonal therapy, targeted therapy, and immunotherapy (8, 40). Chemotherapy plays an imperative role in managing breast cancer, especially for most HER2+ and triple-negative breast cancer patients (8, 41). When selectively killing cancer cells, the healthy tissue damaged simultaneously, causing severe physical burdens for patients such as nausea and vomiting, liver and kidney damage, and so on. That is to say, the current treatments hardly satisfy the requirements of patients. This just provides an opportunity for the development of CHM. In recent years, the application of CHM combined with chemotherapy in cancer treatment has been continuously expanded and well-received by both doctors and patients.

Physicians of the previous dynasties have accumulated rich experience in treating Breast cancer, which belongs to the category of “Ru yan “in TCM. When confronted with cancers, TCM has a distinctive theory of it. Compared to killing tumor cells, TCM puts more attention on maintaining the interior ecology of the body from the perspective of patients’ whole concept. The cornerstone in the management of cancer is to “fu zheng” and “qu xie” in TCM. The so-called “fu zheng” is to strengthen healthy qi, corresponding to improve immunity; the so-called “qu xie” is to remove pathogenic qi, corresponding to killing cancer cells. TCM and Western medicine share the same fundamental concepts when treating cancer. More specifically, breast cancer is mainly located in the liver and spleen. The disharmony of them leads to the occurrence of the disease. Due to the dysregulation of the liver, qi stagnates locally; due to the dysfunction of the spleen in transportation, phlegm and dampness generate. With the progressive nature of disease, a stopping of the fluid flow and qi in the mammary glands contributes to masses, growth, and tumors, eventually developing into breast cancer. Negative emotions are another pathogenic factor for breast cancer, which could lead to a disorder of qi and blood in the liver and spleen, thus resulting in a vicious cycle. XYS has long been used for patients with emotional diseases such as depression, especially for women. The primary function is to soothe the liver and disperse depression, strengthen the spleen and tonify the blood. The whole prescription is simplified, only consisting of 8 herbs. Chai Hu, serving as a monarch drug, disperses the stagnated liver qi to resolve depression, thus the flow of qi becoming normal. Dang Gui nourishes the blood and promotes blood flow. Bai Shao replenishes the yin and blood and softens the liver to relieve pain, acting together as a ministerial drug with Dang Gui. The primary function of Bai zhu and Fu Ling is to strengthen the spleen and supplement qi to transport dampness, and then qi and blood have the source. Summarily, the various components in the whole formula are coordinated and compatible, working together to treat breast cancer.

An increasing number of pharmacological and cellular experiments have demonstrated modified XYS to be of significance in breast cancer. The serum containing Danzhi Xiaoyao Powder can inhibit the proliferation of breast cancer MDA-MB-231 cells, promote cell apoptosis, and block cell cycle (42). Besides, a serum containing Danzhi Xiaoyao San may enhance MDA-MB-231 cell apoptosis, inhibit MDA-MB-231 cell proliferation, invasion, and adhesion, and block MDA-MB-231 cells within the G1 phase by regulating CAF (43). After 4T1-induced ovariectomized Balb/c female mice were treated with Xiaoyao Pills, cell apoptosis was triggered at the protein level, Bcl-2 was considerably reduced, Bax and TP53 protein expressions were significantly increased, and tumor growth was significantly inhibited (44). Further research showed that breast cancer cells MCF-7 and MDA-MB-231 could be prevented from proliferating and migrating by using Longbei Xiaoyao Powder, which may be related to the regulation of the miR-145/c-Myc/TP53 signaling pathway through the miR-145 promoter demethylation and the expression of apoptosis-related genes caspase3, Bcl-2 and Bax (45). XYS had a complex composition, mainly consisting of Kaempferol, saikosaponins, quercetin, ferulic acid, and so on (46, 47). In human breast cancer MDA MB 231 cells, saikosaponins triggered apoptosis by activating the p38 mitogen-activated protein kinase (MAPK) signaling pathway (48). Kaempferol could greatly suppress breast cancer MDA-MB-231 cells, which also induced apoptosis and DNA damage in MDA-MB-231 cells (49). Increased apoptosis was seen in an MDA-MB-231 xenograft mice model, which further supported the anticancer potential of ferulic acid (50). The main molecular targets in research of late were Bcl-2 and Bax, and the critical anticancer mechanism was anti-apoptosis. Human breast cancer cells MDA-MB-231 were focal points lately. To better understand on multi-target intervention of modified XYS against breast cancer, we should uncover potential mechanism further.

Inevitably, this study is subject to some limitations, which should be considered as priorities before applying any conclusions from this study to clinical practice. Firstly, all the studies involved in this review are from China and published in Chinese, making the results difficult to generalize. In other words, there may be some publication bias. We hope to further see more clinical trials reported in English or other languages. Second, the methodological quality of the studies was generally subpar. Only 14 of the recruited studies have mentioned random sequence generation. Allocation concealment in all the studies remains unclear. None of the trials were double-blind. Therefore, several included studies are graded as low quality, resulting in the unclear/high risk of selection bias. This may lead to false positive results. Third, although we have made our search strategy as comprehensive as possible, there may be some omissions. Furthermore, all the included formulas vary from dosage to composition, making it difficult to reach a certain conclusion. In other words, there is still room for improvement. Given the heterogeneity of breast cancer, tumor stratification should be considered, for better precise treatment (51). Last but not least, many active ingredients of CHM have phytoestrogen-like effects. Therefore, the classification of estrogen receptor status of tumors should not be ignored. Hence, future research should be related to specific types, and grade of breast cancer, particularly estrogen receptor status.

In conclusion, we hope the outlined findings will provide clinical physicians with more options. With the increasing application of XYS in cancers, the internationalization of TCM has been greatly facilitated simultaneously. When confronted with breast cancer, more and more clinicians and patients will take XYS into account, which is exactly what we want to see.

## Conclusion

Taken together, the current evidence has revealed that modified XYS combined with chemotherapy certainly has therapeutic effect for patients with breast cancer. However, Considering the limited quality and small sample size, we should be cautious with the results. More double-blinded, large-scale, multi-center, well-designed clinical trials focusing on potential mechanisms need to be conducted further.

## Author contributions

JP contributed conception and design of the study. DL developed the search terms and organized the databases. SF and DL collected the data. JP wrote the draft of the manuscript. SF and JP prepared the figures and tables. JP was the first authorship. QC revised the manuscript. SF and QZ shared the second authorship and contributed equally to this work. All authors contributed to the article and approved the submitted version.
