# Supplementary material for: Modified xiaoyao san combined with chemotherapy for breast cancer: A systematic review and meta-analysis of randomized controlled trials
Source: Front Oncol. 2023 Mar 22;13:1050337. doi: 10.3389/fonc.2023.1050337 (PMC10073574; doi:10.3389/fonc.2023.1050337)
Supplement: Supplementary file 1 [file DataSheet_1.docx]

**Pubmed**

(("Breast Neoplasms"[Mesh]) OR (Breast Neoplasms OR Breast Neoplasm OR Neoplasm, Breast OR Breast Tumors OR Breast Tumor OR Tumor, Breast OR Tumors, Breast OR Neoplasms, Breast OR Breast Cancer OR Cancer, Breast OR Mammary Cancer OR Cancer, Mammary OR Cancers, Mammary OR Mammary Cancers OR Malignant Neoplasm of Breast OR Breast Malignant Neoplasm OR Breast Malignant Neoplasms OR Malignant Tumor of Breast OR Breast Malignant Tumor OR Breast Malignant Tumors OR Cancer of Breast OR Cancer of the Breast OR Mammary Carcinoma, Human OR Carcinoma, Human Mammary OR Carcinomas, Human Mammary OR Human Mammary Carcinomas OR Mammary Carcinomas, Human OR Human Mammary Carcinoma OR Mammary Neoplasms, Human OR Human Mammary Neoplasm OR Human Mammary Neoplasms OR Neoplasm, Human Mammary OR Neoplasms, Human Mammary OR Mammary Neoplasm, Human OR Breast Carcinoma OR Breast Carcinomas OR Carcinoma, Breast OR Carcinomas, Breast)) AND (xiaoyao OR xiaoyao san OR modified xiaoyao san OR xiaoyao powder OR xiaoyao pill OR jia-wei-xiao-yao-san OR jiawei-xiaoyao-san OR xiao yao OR xiao yao san OR xiao yao pill OR xiao yao powder OR smooth liver and strengthen spleen OR smooth liver and regulate qi OR xiaoyao decoction) AND (chemotherapy) AND (randomized controlled trials OR randomization OR randomized OR RCT OR randomly)

**Cochrane Library**

#1 MeSH descriptor: [Breast Neoplasms] explode all trees

#2 (Breast Neoplasm OR Neoplasm, Breast OR Breast Tumors OR Breast Tumor OR Tumor, Breast OR Tumors, Breast OR Neoplasms, Breast OR Breast Cancer OR Cancer, Breast OR Mammary Cancer OR Cancer, Mammary OR Cancers, Mammary OR Mammary Cancers OR Malignant Neoplasm of Breast OR Breast Malignant Neoplasm OR Breast Malignant Neoplasms OR Malignant Tumor of Breast OR Breast Malignant Tumor OR Breast Malignant Tumors OR Cancer of Breast OR Cancer of the Breast OR Mammary Carcinoma, Human OR Carcinoma, Human Mammary OR Carcinomas, Human Mammary OR Human Mammary Carcinomas OR Mammary Carcinomas, Human OR Human Mammary Carcinoma OR Mammary Neoplasms, Human OR Human Mammary Neoplasm OR Human Mammary Neoplasms OR Neoplasm, Human Mammary OR Neoplasms, Human Mammary OR Mammary Neoplasm, Human OR Breast Carcinoma OR Breast Carcinomas OR Carcinoma, Breast OR Carcinomas, Breast)

#3 #1 AND #2

#4 (xiaoyao OR xiaoyao san OR modified xiaoyao san OR xiaoyao powder OR xiaoyao pill OR jia-wei-xiao-yao-san OR jiawei-xiaoyao-san OR xiao yao OR xiao yao san OR xiao yao pill OR xiao yao powder OR smooth liver and strengthen spleen OR smooth liver and regulate qi OR xiaoyao decoction)

#5 (chemotherapy)

#6 (randomized controlled trials OR randomization OR randomized OR RCT OR randomly)

#7 #3 AND #4 AND #5 AND #6 in Trials

**EMBASE**

#1 'breast tumor'/exp

#2 'Breast Neoplasm':ab,kw,ti OR 'Neoplasm, Breast':ab,kw,ti OR 'Breast Tumors':ab,kw,ti OR 'Breast Tumor':ab,kw,ti OR 'Tumor, Breast':ab,kw,ti OR 'Tumors, Breast':ab,kw,ti OR 'Neoplasms, Breast':ab,kw,ti OR 'Breast Cancer':ab,kw,ti OR 'Cancer, Breast':ab,kw,ti OR 'Mammary Cancer':ab,kw,ti OR 'Cancer, Mammary':ab,kw,ti OR 'Cancers, Mammary':ab,kw,ti OR 'Mammary Cancers':ab,kw,ti OR 'Malignant Neoplasm of Breast':ab,kw,ti OR 'Breast Malignant Neoplasm':ab,kw,ti OR 'Breast Malignant Neoplasms':ab,kw,ti OR 'Malignant Tumor of Breast':ab,kw,ti OR 'Breast Malignant Tumor':ab,kw,ti OR 'Breast Malignant Tumors':ab,kw,ti OR 'Cancer of Breast':ab,kw,ti OR 'Cancer of the Breast':ab,kw,ti OR 'Mammary Carcinoma, Human':ab,kw,ti OR 'Carcinoma, Human Mammary':ab,kw,ti OR 'Carcinomas, Human Mammary':ab,kw,ti OR 'Human Mammary Carcinomas':ab,kw,ti OR 'Mammary Carcinomas, Human':ab,kw,ti OR 'Human Mammary Carcinoma':ab,kw,ti OR 'Mammary Neoplasms, Human':ab,kw,ti OR 'Human Mammary Neoplasm':ab,kw,ti OR 'Human Mammary Neoplasms':ab,kw,ti OR 'Neoplasm, Human Mammary':ab,kw,ti OR 'Neoplasms, Human Mammary':ab,kw,ti OR 'Mammary Neoplasm, Human':ab,kw,ti OR 'Breast Carcinoma':ab,kw,ti OR 'Breast Carcinomas':ab,kw,ti OR 'Carcinoma, Breast':ab,kw,ti OR 'Carcinomas, Breast':ab,kw,ti

#3 #1 OR #2

#4 'xiaoyao':ab,kw,ti OR 'xiaoyao san':ab,kw,ti OR 'modified xiaoyao san':ab,kw,ti OR 'xiaoyao powder':ab,kw,ti OR 'xiaoyao pill':ab,kw,ti OR 'jia-wei-xiao-yao-san':ab,kw,ti OR 'jiawei-xiaoyao-san':ab,kw,ti OR 'xiao yao':ab,kw,ti OR 'xiao yao san':ab,kw,ti OR 'xiao yao pill':ab,kw,ti OR 'xiao yao powder':ab,kw,ti OR 'smooth liver and strengthen spleen':ab,kw,ti OR 'smooth liver and regulate qi':ab,kw,ti OR 'xiaoyao decoction':ab,kw,ti

#5 'chemotherapy':ab,kw,ti

#6 randomized controlled trials'

#7 randomization'

#8 randomized'

#9 RCT'

#10 randomly'

#11 #6 OR #7 OR #8 OR #9 OR #10

#12 #3 AND #4 AND #5 AND #11

**Web of Science**

#1 TS=(Breast Neoplasms OR Breast Neoplasm OR Neoplasm, Breast OR Breast Tumors OR Breast Tumor OR Tumor, Breast OR Tumors, Breast OR Neoplasms, Breast OR Breast Cancer OR Cancer, Breast OR Mammary Cancer OR Cancer, Mammary OR Cancers, Mammary OR Mammary Cancers OR Malignant Neoplasm of Breast OR Breast Malignant Neoplasm OR Breast Malignant Neoplasms OR Malignant Tumor of Breast OR Breast Malignant Tumor OR Breast Malignant Tumors OR Cancer of Breast OR Cancer of the Breast OR Mammary Carcinoma, Human OR Carcinoma, Human Mammary OR Carcinomas, Human Mammary OR Human Mammary Carcinomas OR Mammary Carcinomas, Human OR Human Mammary Carcinoma OR Mammary Neoplasms, Human OR Human Mammary Neoplasm OR Human Mammary Neoplasms OR Neoplasm, Human Mammary OR Neoplasms, Human Mammary OR Mammary Neoplasm, Human OR Breast Carcinoma OR Breast Carcinomas OR Carcinoma, Breast OR Carcinomas, Breast)

#2 TS=(xiaoyao OR xiaoyao san OR modified xiaoyao san OR xiaoyao powder OR xiaoyao pill OR jia-wei-xiao-yao-san OR jiawei-xiaoyao-san OR xiao yao OR xiao yao san OR xiao yao pill OR xiao yao powder OR smooth liver and strengthen spleen OR smooth liver and regulate qi OR xiaoyao decoction)

#3 TS=(chemotherapy)

#4 TS=(randomized controlled trials OR randomization OR randomized OR RCT OR randomly)

#5 #1 AND #2 AND #3 AND #4

**China Academic Journal Network (CNKI)**

(SU=’乳腺癌’ OR SU=’乳腺肿瘤’ SU=’乳癌’ OR SU=’乳岩’) AND (SU=’逍遥散’ OR SU=’逍遥散加减方’ OR SU=’加减逍遥散’ OR SU=’疏肝健脾’ OR SU=’疏肝理气’ ) AND (SU=’化疗’) AND (FT=’随机对照试验’ OR FT=’随机对照实验’ FT=’随机对照研究’ OR FT=’RCT’ OR FT=’随机’ OR FT=’随机对照’)

**WanFang Data**

(主题:"乳腺癌" OR 主题:"乳腺肿瘤" OR 主题:"乳癌"OR 主题:"乳岩") AND (主题:"逍遥散" OR 主题:"逍遥散加减方" OR 主题:"加减逍遥散" OR 主题:"疏肝健脾" OR 主题:"疏肝理气) AND (主题:"化疗") AND (全部:"随机对照试验" OR 全部:"随机对照实验" OR 全部:"随机对照研究" OR 全部:"RCT" OR 全部:"随机" OR 全部:"随机对照")

**Chinese science and technology journals database (VIP)**

主题=(乳腺癌 OR 乳腺肿瘤 OR 乳癌 OR 乳岩) AND主题=(逍遥散 OR 逍遥散加减方 OR 加减逍遥散 OR 疏肝健脾 OR 疏肝理气) AND主题=(化疗) AND 任意字段=(随机对照试验 OR 随机对照实验 OR 随机对照研究 OR RCT OR 随机 OR 随机对照)

**Chinese biomedical literature service system (SinoMed)**

#1 "乳腺肿瘤"[不加权:扩展]

#2 (乳腺癌 OR 乳腺肿瘤OR乳癌 OR 乳岩)

#3 #1 OR #2

#4 "加味逍遥散"[不加权:扩展]

#5 "逍遥散"[不加权:扩展]

#6 (逍遥散加减方 OR 加减逍遥散OR疏肝健脾 OR 疏肝理气)

#7 #4 OR #5 OR #6

#8 (化疗)

#9 "随机对照试验"[不加权:扩展]

#10 (随机对照实验 OR 随机对照研究 OR RCT OR 随机对照 OR 随机)

#11 #9 OR #10

#12 #3 AND #7 AND #8 AND #11
